# Adhesive Bonding to Dentin Improved by Polymerizable Cyclodextrin Derivatives

**DOI:** 10.6028/jres.114.002

**Published:** 2009-02-01

**Authors:** Rafael L. Bowen, Gary E. Schumacher, Anthony A. Giuseppetti, Charles M. Guttman, Clifton M. Carey

**Affiliations:** Paffenbarger Research Center, American Dental Association Foundation, Gaithersburg, MD 20899-8546; Polymers Division National Institute of Standards and Technology, Gaithersburg, MD 20899-0001; Paffenberger Research Center, American Dental Association Foundation, Gaithersburg, MD 20899-8546

**Keywords:** bond strengths, cross-linking comonomers for polymerizable cyclodextrin derivatives, dental adhesive bonding formulations

## Abstract

The objective of this work was to determine bonding characteristics of a hydrophilic monomer formulation containing polymerizable cyclodextrin derivatives. The hypothesis was that a formulation containing hydrophilic cross-linking diluent comonomers and cyclodextrins with functional groups attached by hydrolytically stable ether linkages could form strong adhesive bonds to dentin. The previously synthesized polymerizable cyclodextrin derivatives were formulated with sorbitol dimethacrylate, methacrylic acid and phenylbis(2,4,6-trimethylbenzoyl) phosphine oxide photoinitiator. The same formulation without the polymerizable cyclodextrin derivatives isolated the effects of the polymerizable cyclodextrin derivatives. A commercial self-etching bonding system was tested as a comparative control. Ground mid-coronal dentin was etched with 37 % phosphoric acid (H_3_PO_4_) for 15 s and rinsed with distilled water for 10 s. Formulations were applied to the moist dentin and light-cured 10 s. A packable composite was then applied through irises and light-cured 60 s. Teeth were stored in water for 24 h before bonds were tested in a shearing orientation. One-way ANOVA was performed on the data. The average values of shear bond strengths were defined as loads at fracture divided by the 4 mm diameter iris areas. The average value of shear bond strength for the formulation containing the polymerizable cyclodextrin derivatives was higher (p < 0.05), where p is a fraction of the probability distribution) than that of the same monomeric formulation except that the polymerizable cyclodextrin derivatives were not included. This was supporting evidence that the polymerizable cyclodextrin derivatives contributed to improved bonding. The average value of shear bond strength for the formulation containing the polymerizable cyclodextrin derivatives was also higher *(p* < 0.05) than that of the commercial self-etching bonding system. These preliminary results are in accordance with the hypothesis that formulations containing polymerizable cyclodextrin derivatives can form strong adhesive bonds to hydrated dentin surfaces. Further improvements in bonding to hydrated biological tissues by use of advanced formulations are anticipated.

## 1. Introduction

Because most dentists spend much of their time restoring teeth that have developed what appear to be recurrent caries at the margins of previous restorations [1], improved bonding materials and application methods are needed to restore teeth esthetically in a manner that will prevent formation of staining and other interfacial defects. It appears that none of the currently available adhesive compositions and their instructions for use is ideal for bonding caries-preventive resins or contemporary composite restorative materials to dental enamel and, especially, to dentin [2]. Replacements of sealants and composite restorations are most often a result of interfacial separations between tooth surfaces and the applied resins, with resulting discolorations and the effects of harbored microorganisms. The present report describes the testing of novel materials with the objective of providing improvements in bonding capabilities.

**Fig. 1:**
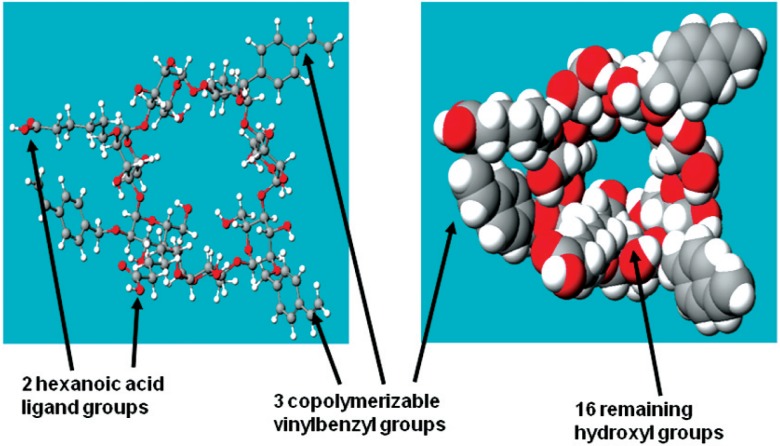
Computer simulation of a polymerizable cyclodextrin derivative (PCD) with a ball-and-stick depiction on the left and a space-filling image on the right. Gray corresponds to carbon atoms, white to hydrogen atoms, and red to oxygen atoms.

**Fig. 2:**
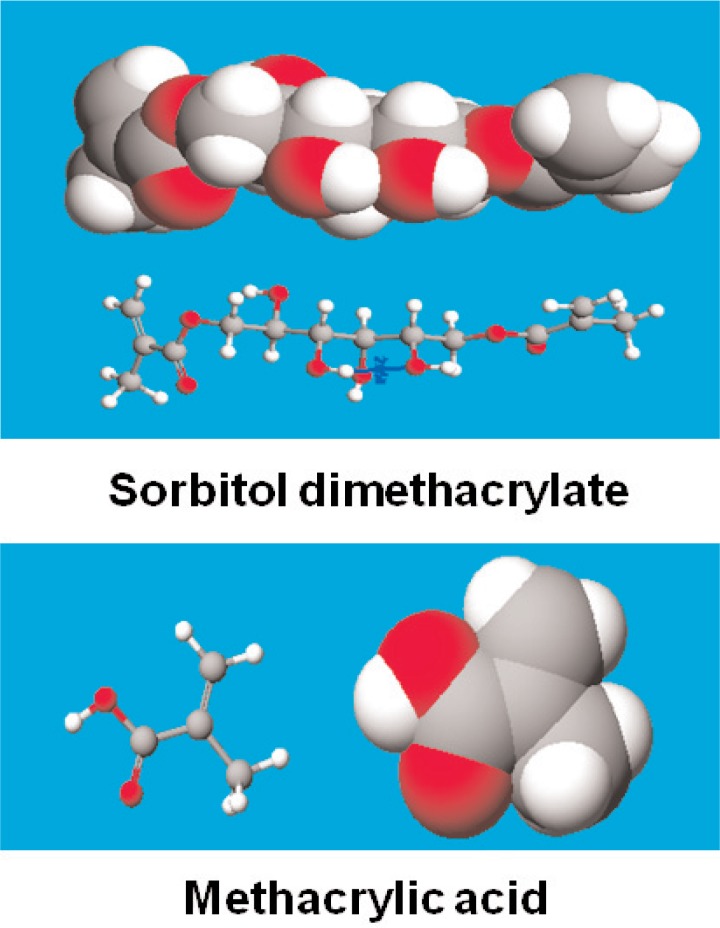
Sorbitol dimethacrylate and methacrylic acid reactive diluents used in the composition of bonding formulations one and two. Gray corresponds to carbon atoms, white to hydrogen atoms, and red to oxygen atoms.

One of the formulations evaluated here includes a family of monomers referred to as polymerizable cyclodextrin derivatives [3,4]. Most of the diverse members of this family each contain polymerizable groups, carboxylate-terminated ligand (surface-binding) groups, and residual hydroxyl groups (Fig. 1.). The hydrophobic copolymerizable groups are posited to copolymerize with diluent comonomers and other resin-based materials. The polymerizable cyclodextrin derivatives’ hydrophilic carboxylate ligand groups can form ionic, surface-binding interactions with the embedded dental collagen and also with the calcium phosphate phases of dentin and enamel. Their residual hydroxyl groups will form electrostatic hydrogen bonding not only with dental substrate sites, but also with their hydrophilic diluent comonomers (Fig. 2.) and with free and bound water molecules remaining in acid-etched tooth surfaces. Due to the hydrophilic ligand and hydroxyl groups, these molecules should be able to penetrate the hydrated layers of dentin and enamel and interact with collagen and tooth mineral. In this study, an attempt was made to have the solubility parameters of Type I collagen, the polymerizable cyclodextrin derivatives, and the diluent comonomers as nearly the same as possible so that the formulation components would not undergo phase separation during impregnation of the hydrated tooth components.

It is (or should be) common practice now to remove mechanically weak surface layers on teeth (“smear layers” and/or salivary pellicle) with an etching procedure. Smear layers can contain microorganisms and can impede the filling and sealing of dentinal tubules and their anastomosing or interconnected lateral canals with cross-linked adhesive polymers [5]. “Total etching” [6] usually comprises the brief application of an acidic solution, such as aqueous phosphoric acid, to remove debris from the tooth surfaces that require coverage with a dental resin. After this etching and a water rinse, the enamel has a superficially porous hydrated surface layer; the surface of dentin consists primarily of a thin layer of hydrated Type I collagen fibrils, somewhat denatured, that are retained by partial embedment in calcium phosphate minerals.

The wet porous enamel and the hydrated collagen network are the substrates to which adhesion must be achieved if good bonding is to be expected. Success strongly depends on the following factors: Complete and simultaneous interpenetration of all of the adhesion-promoting formulation components into the hydrated pores of enamel and throughout the water-filled intact or denatured fibrillar collagen network of dentin and cementum, including interaction with underlying mineral. It seems reasonable to assume that the stoichiometric and physical forms of the original mineral(s) could have been changed by the acidic debridement and/or exposure to acidic monomers. Furthermore, there must be successful displacement of most of the water, by virtue of strong physicochemical affinity of the adhesion-promoting molecules, from the diverse “receptor sites” of these substrates. Formulations containing polymerizable cyclodextrin derivatives such as are reported here (especially improved versions thereof) are good candidates for fulfilling these requirements.

Cyclodextrins, especially appropriate derivatives thereof, comprise cyclic oligomers of glucose that can form water-soluble inclusion complexes with small molecules and portions of large compounds [7]. The synthesis of the polymerizable cyclodextrin derivatives used in this report is described elsewhere [4]. Briefly, these polymerizable cyclodextrin derivatives comprise *beta*-cyclodextrin molecules derivatized to attach combinations and permutations of both hydrophobic (organophilic) polymerizable groups and hydrophilic ligand groups on the members of a “family” of molecules. These molecules, with the groups attached by hydrolytically stable ether linkages, are based on cyclic oligosaccharides of seven glucopyranose units with alpha 1–4 glycoside linkages. The three-dimensional structures of *beta*-cyclodextrin molecules resemble truncated cones with hydrophobic interiors and hydrophilic exteriors that can form water-soluble inclusion complexes with small molecules and portions of large compounds [7]. Polymerizable cyclodextrin derivatives are water soluble because of the hydrophilic ligand groups and what remain of the original 21 hydroxyl groups that did not become derivatized. Although these quasi-spheroidal molecules have molecular weights mostly in the range of 1400 to 2000 grams per mole, three-dimensional computer modeling shows that they have diameters approximately the same as the cross-section of a single triple-helical collagen molecule (in the neighborhood of about 2 nm as estimated from the computer modeling). This is much smaller than the sizes of the observable pores in the surfaces of acid-etched enamel, the internal diameters of dentinal tubules or their lateral canals, or the spaces between the collagen fibrils presenting in intertubular dentin after acid-etching and rinsing with water, according to observations made by staff members here.

The hypothesis in this study is that these types of polymerizable cyclodextrin derivative molecules, formulated with hydrophilic cross-linking diluent comonomers, can penetrate the hydration layers and form stronger adhesive bonds to hydrated dentin when compared with the control formulations.

## 2. Materials and Methods

### 2.1 Shear Bond Adhesion Test Method

Flat dentin surfaces were prepared by cutting off, with running water as a coolant, the tips of crowns of caries-free human molars with a low-speed diamond saw (Isomet; Buehler Ltd., Lake Bluff, IL, USA). The teeth were embedded with cold-curing resin in poly-carbonate holders, and the mid-coronal dentin surfaces were ground perpendicular to the long axis of the tooth on water-washed No. 320 grit SiC paper until the occlusal enamel was completely removed. For all except the commercial control bonding formulation, the dentin surfaces were etched (37 % phosphoric acid gel, Kerr Gel Etchant) for 15 s and rinsed with distilled water for 10 s. Excess water was patted off with a damp cellulose tissue to prevent drying completely.

The sources for the bonding formulation components that were used are given in Table 1. Formulations were applied with a brush-tipped applicator for 20 s, blown “dry” with an air stream for 10 s resulting in a glossy finish. The surfaces were light cured for 10 s with a halogen-type light (Dentsply Caulk Spectrum™ Curing Light). A stainless-steel ring having a having a 4 mm diameter central iris opening was held against the treated surface and a composite material (Prodigy Unidose P-C2, item No. 25886, Exp. 0498, “505096”) was condensed into the opening, with care taken to not overlap the top of the iris. The composite was light-cured for 60 s and then the assembly was left to stand for 5 min, to allow for cooling after the exothermic polymerization of the monomers, before the holder was removed. Then the iris containing the bonded composite was soaked in distilled water for 24 h at about 23 °C before bond testing in the shear mode.

A holding device was used to evaluate the shear bond strengths. The stainless-steel ring containing the dentin-bonded composite within its iris was placed against a vertical surface of a nylon block. The ring and the composite were sheared off, at a crosshead speed of 0.5 mm/min, with a flat chisel pressing against the edge of the steel ring close to the tooth surface. The flat chisel was connected to the platen of a Universal Testing Machine (Instron Corp., Canton, MA).

### 2.2 Experimental Bonding Formulation 1

**Fig. 3:**
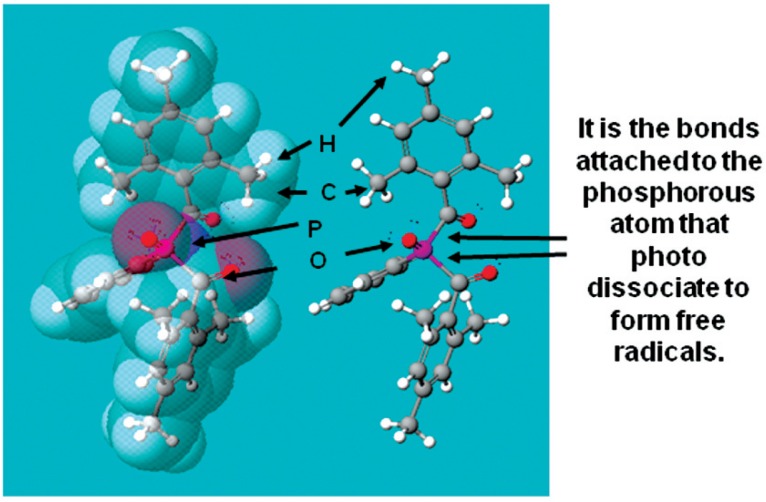
Photoinitiator used in the experimental formulations: phenyl bis(2,4,6-trimethylbenzoyl) phosphine oxide. These molecules are too large to become totally complexed (“cloistered,” encapsulated) within the hydrophobic core spaces of polymerizable cyclodextrin derivatives. However, in an increasingly aqueous environment during penetration of surface hydration layers, the aromatic side groups would be expected to become sufficiently complexed to become surrounded by hydrophilic PCD molecules to enable diffusion and accompaniment of the comonomers to reach the unaltered substrate minerals. Gray corresponds to carbon atoms, white to hydrogen atoms, red to oxygen atoms and purple phosphorous atoms.

In the experimental formulation 1, a portion (0.0419 g) of the family of polymerizable cyclodextrin derivative reaction products [3, 4] was mixed with sorbitol dimethacrylate (0.3261 g “SDM2”) and methacrylic acid (0.2383 g MAA) as reactive diluents to lower the polymerizable cyclodextrin derivative viscosity. Phenylbis[2,4,6-trimethylbenzoyl]phosphine oxide (0.0271 g Irgacure^®^ 819) was added to serve as the photoinitiator (Fig. 3.) to induce polymerization. The formulation also contained a very small amount of the antioxidant Irganox^®^ 1330 plus the stabilizers contained in the sorbitol dimethacrylate and methacrylic acid as received. The composite was applied and the shear bond adhesion test method for Formulation 1 was performed as described in Sec. 2.1, with *n* = 6, where *n* is the number of independent samples used for the shear bond strength measurements.

### 2.3 Comparative Bonding Formulation 2

The same formulation 1 without the family of polymerizable cyclodextrin derivative reaction products, maintaining the photoinitiator concentration approximately constant, was also prepared to isolate the effect of the presence of the polymerizable cyclodextrin derivatives. This formulation contained 0.3261 g of SDM2, 0.2383 g of methacrylic acid, 0.0271 g of Irgacure^®^ 819, and the same small amount of Irganox^®^ 1330 plus the stabilizers contained in the SDM2 and methacrylic acid as received. The composite was applied and the shear bond adhesion test method was the same as that described in Sec. 2.1, with *n* = 9.

### 2.4 Control Bonding Formulation 3

A commercially-available self-etching primer bonding system was used as a control (Clearfil Protect Bond Primer No. 1, lot 0004A, and Clearfil Protect Bond, Kuraray Med. Inc., Kurashiki, Okayama, Japan, Lot 00008A). Components listed in a Material Safety Data Sheet for “Bond Liquid, Clearfil Protect Bond” are as follows: “Silanated colloidal silica, sodium fluoride, bisphenol A diglycidylmethacrylate, 2-hydroxyethyl methacrylate, hydrophobic dimethacrylate, 10-methacryloyloxydecyl dihydrogen phosphate, N,N-diethanol-p-toluidine, and d,l-camphorquinone” [8]. The manufacturer’s instructions were followed: The abraded dentin surface was rinsed with distilled water for 10 sec and then excess water was patted off with a damp cellulose tissue without completely drying. The self-etching primer was gently applied, mildly dried with the airflow, and then covered with the Protect Bond. The solvent was removed with the airflow, and then the coated surface was light-cured for 10 s. The composite was placed as described in Sec. 2.1 and light-cured for 60 s. The shear bond adhesion test method was also the same as that described in Sec. 2.1, with *n* = 6.

### 2.5 An Alternative Bonding Procedure With Formulation 1

An exploratory comparison was made with the experimental formulation 1 (B161-136 family of polymerizable cyclodextrin derivatives plus sorbitol dimethacrylate plus methacrylic acid plus Irgacure^®^ 819, with the difference that after the etching, rinsing, and removal of excess water with a damp cellulose tissue, 200-proof ethanol was applied to the dentin surface with a brush-tipped applicator for 10 s to replace much of the surface water with alcohol before application of the experimental formulation [9]. Otherwise, the procedure was as in Sec. 2.1, with *n* = 3.

### 2.6 An Alternative Bonding Procedure With Formulation 2

The formulation and testing procedure were the same as in Sec. 2.3, without the family of polymerizable cyclodextrin derivative reaction products and maintaining the photoinitiator concentration approximately constant, except that after the excess water was removed from the surfaces, 200-proof ethanol was applied to the dentin surfaces with a brush-tipped applicator for 10 s to replace much of the surface water with alcohol [9]. The shear bond adhesion test method was the same as that described in Sec. 2.1, with *n* = 3.

### 2.7 Another Alternative Bonding Procedure With Formulation 1

This comparison was made as described in Sec. 2.1 with the differences being that after the excess water was patted off, 200-proof ethanol was applied to the dentin surfaces with a brush-tipped applicator for 10 s to replace much of the surface water with alcohol [9], the experimental formulation (B161-136 family of polymerizable cyclodextrin derivatives plus sorbitol dimethacrylate plus methacrylic acid plus Irgacure^®^ 819) as described in Sec. 2.2 was applied with a brush-tipped applicator for 20 s, blown “dry” with an air stream for 10 s resulting in a glossy finish. Additionally, this was then covered with Clearfil Protect Bond. The solvent was removed with the airflow, and then the coated surface was light cured for 10 s. Otherwise, the procedure was as in Sec. 2.1, with *n* = 3.

### 2.8 Statistics

One-way analysis of variance (ANOVA) was performed on the results to detect significant effects. Newman-Keuls multiple comparison tests were conducted with the data of Secs. 2.2, 2.3, and 2.4 to determine if a significant difference (*p* < 0.05) existed where *p* is a fraction of the probability distribution.

### 2.9 Scanning Electron Microscopy

After the shear bond adhesion tests, some of the teeth were cut longitudinally and treated to evaluate the hybrid layer. Two methods were used: 30 % phosphoric acid etching for 15 s and bleaching with 6.15 % sodium hypochlorite (Ultra Clorox^®^) for 8 min, or polishing and argon-ion etching for 5 min.

## 3. Results

### 3.1 Adhesion Tests

The shear bond strength average values, defined as the load at fracture divided by the iris opening areas, are given in Table 2. The average shear bond strength of formulation 1 (Sec. 2.2) containing the family of PCD reaction products was significantly higher (*p* < 0.05) than that of formulation 2 (Sec. 2.3), which was the same formulation minus the family of PCD reaction products, indicating that the presence of polymerizable cyclodextrin derivatives led to improved bonding. The average shear bond strength of formulation 1 (Sec. 2.2) ranked higher than that of formulation 3, the commercial control (Sec. 2.4). The preliminary application of alcohol in procedure Sec. 2.5 did not increase the average bond strength with formulation 1 in comparison with that described in Sec. 2.2. The preliminary application of alcohol in procedure Sec. 2.6 with formulation 2 did not increase the average bond strength relative to formulation 1 in procedure Sec. 2.2, but its mean bond strength ranked higher than its value with procedure Sec. 2.3. Procedure Sec. 2.7 did not give an increase in average bond strength relative to that obtained with procedure Sec. 2.2.

### 3.2 Statistics

One-way ANOVA analysis found that the type of formulation used had a significant effect (*p* < 0.05) on the shear bond strength values obtained with procedures Sec. 2.2 and Sec. 2.3.

### 3.3 Scanning Electron Microscopy

**Fig. 4:**
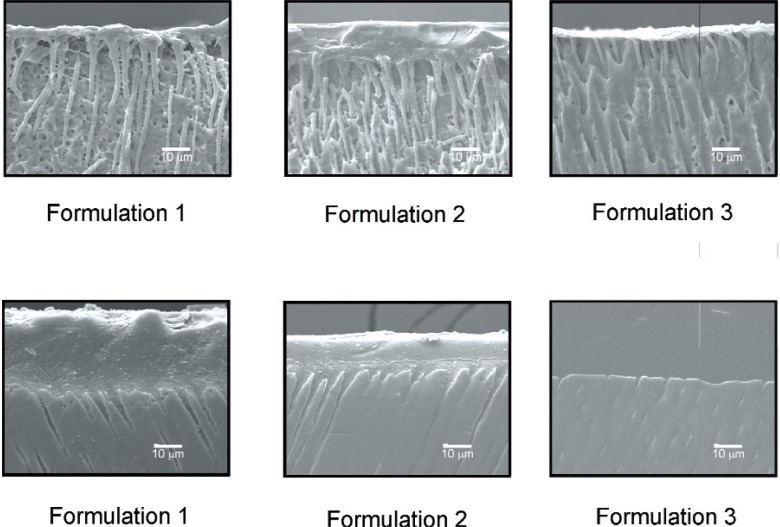
SEM pictures of debonded teeth: These pictures showed evidence of hybrid layer formations in the case of Formulations 1 and 2. For the control (Formulation 3) a hybrid layer could not be detected. The upper image of the Formulation 1 sample shows continuity of the hybrid layer with the polymer in the tubules and in the lateral canals. The upper images are of samples that had been acid etched for 15 s and bleached for 8 min. The lower images are of samples that had been argon-etched. The white bars represent 10 micrometers.

The formulation 1 containing the derivatized *beta*-cyclodextrins (2.2) and the formulation 2 not containing the family of polymerizable cyclodextrin derivatives (2.3) resulted in mostly cohesive failures within the resinous materials, with the areas of separation approximately the same as the areas of the iris opening, as observed with a binocular measuring microscope (Model MZ16 Stereo-optical microscope system, with traveling stage, by Leica Microsystems, Wetzlar, Germany). The commercial control formulation 3 (described in Sec. 2.4) failed mostly at its adhesive interface with dentin (Fig. 4), and the areas of adhesive separation tended to be larger than the areas of the iris opening.

## 4. Discussion

Reported shear bond strength values vary widely, depending in part on the selected test procedures [10]. Therefore, numerical values should be compared only within the method that is used.

Although only about a maximum of seven hydroxyl groups in molecules of this family of polymerizable cyclodextrin derivatives were substituted, the bonding formulation prepared with this family of reaction products did result in an average shear bond strength significantly higher than that of the analogous formulation that did not contain this family of polymerizable cyclodextrin derivatives. One of the important distinctions of this polymerizable cyclodextrin derivative family was that the synthetic procedure attached both the polymerizable groups and the terminal carboxylate ligand groups to the *beta*-cyclodextrin molecules with hydrolytically stable covalent ether linkages. There is reason to believe that some of the instability associated with contemporary adhesive resins is related to hydrolysis and/or saponification of ester linkages within the interfacial bonding polymers [11, 12]. For reasons of expediency, the diluent cross-linking comonomer sorbitol dimethacrylate was used in the present study even though it contained potentially hydrolyzable ester linkages. The preparation and evaluation of “sorbitoldivinylbenzyl ether” is planned for use as an alternative.

Another important distinction was that the vinylbenzyl groups should have facile free-radical polymerization charactistics similar to that of styrene. The vinyl double bonds of styrene readily homopolymerizes and copolymerizes with the double bonds of methacrylate esters. However, the stable covalent attachment of the vinylbenzyl groups to such large hydrophilic molecules as polymerizable cyclodextrin derivatives should obviate the biological incompatibility of styrene, as such, in dental formulations. However, it is essential, of course, that all new formulations, including the novel compositions described herein be tested and evaluated in a manner that assures their safety for use in dental or bio-medical applications.

This study does not address the intimately related problem of the polymerization shrinkage stresses that develop primarily during the cooling that follows the exothermic polymerization of composite materials and other dental resins [13–17]. However, this study together with the investigation results of many others have introduced novel concepts for modifications of compositions relevant to lessening the stresses that composites and adhesion-promoting formulations currently generate at tooth-polymer interfaces. Investigation of these concepts is planned.

## 5. Conclusions

These preliminary results [18] are in accordance with the hypothesis that polymerizable cyclodextrin derivative molecules, formulated with hydrophilic cross-linking diluent comonomers, can penetrate the hydration layers and form strong adhesive bonds to hydrated dentin as compared to the controls. The hypothesis was supported. However, improved synthetic methods are underway to obtain more than one polymerizable group on substantially every reaction product molecule to provide densely cross-linked polymers, and to obtain an increased number of ligand groups on these molecules to enable an increased number of binding interactions with substrate “receptor sites.” It is important that the water-soluble polymerizable cyclodextrin derivative molecules can transport water-insoluble polymerization initiators by means of complexation, together with the hydrophilic cross-linking diluent comonomers, through surface hydration layers to form strong adhesive bonds.

## Figures and Tables

**Table 1:** Materials used in the polymerizable cyclodextrin derivative (PCD) adhesive-bonding formulation

Acronym	Chemical	Lot	Manufacturer.
PCDs	Family of polymerizable cyclodextrin derivatives	B161-12	Synthesized
Irganox^®^ 1330	1,3,5-trimethyl-2,4,6-tris(3,5-di-(tert)-butyl-4-hydroxybenzyl)benzene	11107	Ciba Specialty Chemicals Corp., Tarrytown, NY 10591
SDM2	sorbitol dimethacrylate	9676 18-6-1	Monomer-Polymer & Dajac Laboratories, Inc. Feasterville, PA 19053
MAA	methacrylic acid	09610HB	Sigma-Aldrich, Inc., St. Louis, MO 63103
Irgacure^®^ 819	Phenyl bis-(2,4,6-trimethylbenzoyl)-phosphine oxide	21J887S	Ciba Specialty Chemicals Corp., Tarrytown, NY 10591

**Table 2:** Shear bond strength (SBS) average values in MPa (where 1 Pa = 1 kg · m^−1^ · s^−2^), defined as the load at fracture divided by the iris opening areas. The estimates of standard uncertainty are indicated by the standard deviation (SD) values.

*Formulation* *Procedure*	*1* *2.2* (*n* = *6*)	*2* *2.3* (*n* = *9*)	*3* *2.4* (*n* = *6*)	*1* *2.5* (*n* = *3*)	*2* *2.6* (*n* = *3*)	*1* *2.7* (*n* = *3*)
SBS in MPa (SD)	24.6 (5.4)	12.4 (3.7)	17.4 (4.0)	22.7 (4.6)	19.2 (3.8)	22.7 (6.7)
